# Autologous Blood Injection for Treatment of Tennis Elbow

**DOI:** 10.5812/traumamon.5095

**Published:** 2013-01-15

**Authors:** Mahmood Karimi Mobarakeh, Ali Nemati, Ali Fazli, Amirhossein Fallahi, Saeid Safari

**Affiliations:** 1 Department of Trauma and Orthopedic Surgery, Kerman University of Medical Sciences, Shahid Bahonar Hospital, Kerman, IR Iran; 2 Department of Trauma and Orthopedic Surgery, Shahid Beheshti University of Medical Sciences, Imam Hossein Hospital, Tehran, IR Iran; 3 Department of Emergency Medicine, Shahid Beheshti University of Medical Sciences, Shohada Hospital, Tehran, IR Iran

**Keywords:** Tennis Elbow, Blood injection, Autologous

## Abstract

**Background:**

Tennis elbow (TE) is a common myotendinosis. It was first described by Runge in 1873; different modes of treatment are used in management of TE.

**Objectives:**

This study aimed to report the results of autologous blood injection (ABI) in the treatment of TE.

**Materials and Methods:**

A prospective case study was performed to evaluate the results of ABI in the management of TE. The level of pain based on Nirschl phase scale (NPS) and a visual analogue scale (VAS) was calculated before and 1, 3 and 6 months after injection; then satisfaction was assessed.

**Results:**

Twenty-nine patients with diagnosed TE were treated by ABI (24% males, 76 % female). The mean age of the patients was 44.1 ± 5.2 years. The level of pain on VAS decreased from 6.46 ± 2.08 to 0.54 ± 0.7 (P=0.001) and on NPS from 6.15 ± 1.48 to 0.54 ± 0.76 (P = 0.001) 6 months after treatment. At the end of the study, 84% of patients expressed a high level of satisfaction.

**Conclusions:**

Given the acceptable outcomes, autologous blood injection can be considered a good treatment option for TE when traditional treatment has fails.

## 1. Background

Tennis elbow (TE) is a common myotendinosis first described by Runge in 1873 (1). It is a clinical diagnosis verified via ultrasonography and magnetic resonance imaging (2, 3). There are a number of treatment modalities such as repetitive low-energy shock wave (4), physical therapy (5) and open surgical treatment (6) for management of TE. However, no treatment method has shown to be superior to others. One of the common treatments is the injection of corticosteroid (7). The logic behind its use is based on the theory that the disease is inflammatory. Recent studies demonstrate that TE is a proliferative process and it is named angiofibroblastic degeneration or hyperplasia. In 1993 Edwards and Calandruchio published their paper regarding use of autologous blood in treatment of TE even in those patients that were not cured by other methods (8).It is stated that blood contains humeral and cellular mediators that initiate an inflammatory process in the injured tissue and result in repair.

## 2. Objectives

This study aimed to evaluate the results of ABI in treatments of TE.

## 3. Materials and Methods

In the series of prospective case studies, 29 patients referred to the orthopedic clinic of our hospital with TE and were treated by ABI. Comprehensive verbal and written explanations were given to the patients regarding different methods of treatment; all patients gave informed consent prior to being included into the study. Patients with a history of surgery on the injured elbow were excluded. The study was authorized by the local ethical committee and was performed in accordance with the Ethical standards of the1964 Declaration of Helsinki as revised in 2000. Patients were clinically examined before injection; 2 ml of autologous blood taken from the ipsilateral upper extremity vein plus 1ml of Lidocaine 2%was injected at the point of maximal pain. Immobilization via a long arm cast was done for 3 weeks. The level of pain based on NPS and VAS was measured 1, 3 and 6 months after injection. At the end of the study the satisfaction level of the patients was measured by Verhaar scale. All patients were treated by the same surgeon. In cases of equivocal clinical findings and to differentiate the radial tunnel syndrome, EMG/NCV was performed (11 patients).

## 4. Results

Twenty-nine patients with recognized TE treated by ABI were studied (24% male, 76% female). Three patients were omitted from the study due to unavailability for follow-up. The mean age of patients was 44.1± 5.2 years (range: 25-68). In 19 (65%) patients the right hand was affected (75% in dominant hand). The mean duration of symptoms was 7.9 ± 1.3 months. Table 1 shows results of NPS and VAS before and 1, 3 and 6 months after ABI. Mean NPS before and after 6 months of treatment was 6.15 ± 1.48 and 0.54 ± 0.76 respectively (P = 0.001). Also the mean level of VAS before and after 6 months of injection was 6.46 ± 2.08 and 0.54 ± 0.7 respectively (P = 0.001). The level of patient satisfaction on Verhaar scale is shown in Figure 1; 84% of patients showed a high level of satisfaction at the end of the study. None required a second injection, although some of them were obliged to change their activities. No infection after injection was detected. In 2 patients, skin color change was observed.

## 5. Discussion

According to the results of the current study, our patients experienced significant reduction in pain level 6 months after ABI. Also 85% of injured patients had a high level of satisfaction at the end of the study. The patients’ age was similar to other comparable studies (1, 8, 9). The level of pain severity based on VAS and NPS at the first visit, before initiation of treatment, was low in comparison with similar studies. The level of pain reduction on VAS and NPS after 3, and 6 months was comparable to previous studies. Also the level of satisfaction was similar to other studies (9, 10).

According to previous studies similar treatment has been done for tennis elbow: corticosteroid injection, corticosteroid iontophoresis, shock wave therapy, and percutaneous tennis elbow release under local anaesthesia have also been used (6, 7, 11, 12).In similar studies Percutaneous release of the epicondylar muscles for humeral epicondylitis has been done as a viable treatment option after the failed conservative management of tennis elbow(12). In other studies corticosteroid injections have shown a marked short-term effect on pain (7).

Our study showed ABI can be considered a low cost treatment method for TE when traditional treatment has failed.
